# Intraoperative differentiation of pancreatic neoplastic lesions using optical coherence tomography (OCT)

**DOI:** 10.1007/s00423-025-03810-9

**Published:** 2025-07-18

**Authors:** Markus Kist, Paul Strenge, Tobias Keck, Andreas Weber, Peter Bronsert, Thaer S. A. Abdalla, Ulrich Friedrich Wellner, Michael Thomaschewski

**Affiliations:** 1 Department of Surgery, University Medical Center Schleswig-Holstein, Campus Lübeck, Ratzeburger Allee 160, 23538 Lübeck, Germany; 2 Medical Laser Center Lübeck GmbH, Peter-Monnik-Weg 4, 23562 Lübeck, Germany; 3 Core Facility for Histopathology and Digital Pathology, Medical Center- University of Freiburg, 79106 Freiburg, Germany

**Keywords:** Optical coherence tomography, PanNET, IPMN, PDAC

## Abstract

**Purpose:**

The diagnostic methods for accurately differentiating the dignity of pancreatic neoplasms are limited. Worrisome features on MRI and endosonography guide the way to resection or conservative treatment with a relevant rate of failure. Intraoperative minimal invasive optical coherence tomography could be a solution for this challenge. The aim of this study is to investigate whether optical coherence tomography is suitable for differentiating of pancreatic neoplastic lesions.

**Methods:**

In this exploratory study, four patient’s specimens of pancreatic resections (white adipose tissue, intraductal papillary mucinous neoplasm (IPMN), pancreatic ductal adenocarcinoma (PDAC) based on IPMN and neuroendocrine pancreatic carcinoma) were prospectively examined ex vivo immediately after resection in the operating room using an optical coherence tomography system (Callisto 930nm, Thorlabs GmbH). In detail, the study investigated whether and in what way endocrine tumors, adenocarcinomas, premalignant and benign cysts differ morphologically in optical coherence tomography imaging compared to healthy pancreatic tissue. The final histopathological findings of the pancreatic specimens served as a reference and were correlated.

**Results:**

The samples examined ranged from typical fatty tissue, intraductal papillary mucinous neoplasm (IPMN), a moderate differentiated (G2) pancreatic ductal adenocarcinoma (PDAC) based on an intraductal papillary mucinous neoplasm (IPMN) and a neuroendocrine pancreatic carcinoma. Optical coherence tomography was feasible to replicate key histological characteristics and tissue architecture in correlation to conventional Hematoxylin-eosin histology.

**Conclusion:**

Optical coherence tomography imaging has the potential to differentiate between benign, pre-malignant and malignant pancreatic pathologies by morphology and should be examined in larger collectives.

## Introduction

Currently, the diagnostic methods for precisely differentiating the dignity of pancreatic tumors, especially pancreatic cystic lesions, are limited [1, 2]. Various international research efforts are currently focusing on the early detection of precursors of pancreatic cancer in these entities and define candidates for resection before transition to a malignant state. The diagnostic tools currently used range from the identification of new laboratory chemical biomarkers to imaging techniques [3, 4]. Low-risk pancreatic cystic entities include simple cysts, pseudocysts and serous cystadenomas (SCA). These entities do not require surgical resection if asymptomatic [3, 5]. High-risk entities are mucinous cystic neoplasms (MCN), intraductal papillary mucinous tumors (IPMN) especially with worrisome features and tumors combined of solid and cystic components [3, 6]. Depending on the degree of dysplasia, high-risk entities in classic histologic examination as H&E histology are classified as adenomas, borderline tumors, carcinoma in-situ (CIS) or invasive tumors. Surgical removal is indicated for the high risk entities [5–7].

There are currently three main diagnostic methods available for morphological differentiating pancreatic lesions. These include conventional computed tomography (CT), magnetic resonance imaging (MRI) and endoscopic ultrasound (EUS). However, these diagnostic methods are limited by their resolution to provide adequate information and in the latter by access to the complete organ [8, 9].

Previous studies have identified optical coherence tomography (OCT) as a possible means of live intraoperative histologic examination [9]. OCT is an imaging technique that enables a high optical resolution of in-vivo tissue and thus a precise characterization of cell clusters and the surrounding connective tissue. It is an interferometric measurement method for creating depth section images (B-scans). The characteristics of the depth section images are similar to ultrasound, but OCT is contact-free and is therefore particularly suitable for imaging sensitive tissue. By using light in the near-infrared range, penetration depths of up to 4 mm can be achieved with a resolution of a few micrometers. Another advantage is the high speed of data acquisition, with images and data available for evaluation within a few seconds [9–13] enabling intraoperative use of this technology.

The primary purpose of this study is to investigate whether OCT is suitable for intraoperative differentiating pancreatic neoplastic lesions.

## Materials and methods

### Study population

The present study represents a monocentric prospective explorative study. Recruitment took place from 01.01.2022 to 15.06.2023 after which the evaluation took place. The study population included four patients with a pancreatic lesion requiring surgery. Each of the four patients contributed a distinct type of pancreatic tissue: (a) white adipose tissue, (b) intraductal papillary mucinous neoplasm (IPMN), (c) pancreatic ductal adenocarcinoma (PDAC) based on IPMN, and (d) neuroendocrine pancreatic carcinoma. The presentation of the four patients and the examination of the indication for surgery was carried out independently of the study at the Department of Surgery, University Medical Center Schleswig-Holstein (UKSH, Campus Lübeck). All patients had unclear pancreatic head tumors with CT or MRI morphology “worrisome features” and underwent pylorus-preserving pancreaticoduodenectomy procedure. Demographically, all patients (one male and three female) were fair-skinned and originally from Germany. Mean age was 72 years. Informed consent for the scientific use of the tissue samples and the use of pseudonymized patient data was obtained from the patients preoperatively. The verbal detailed information is followed by the written consent of the patients for the use of the patient-specific biomaterials and research purpose. The ethics committee of the University of Lübeck, Schleswig-Holstein, Germany, has given a positive vote on the use of the samples, associated data and research purpose within the UKSH and the University of Lübeck (#2023 − 362). No minors were included in this study.

### Data collection

In this OCT study, histopathologic specimen were prospectively examined ex vivo immediately after resection in the operating room. For this purpose, pancreatic specimen were imaged with a spectral domain OCT system (Callisto 930 nm, Thorlabs) with an axial resolution of 7 μm. The system is equipped with a LSM03-BB objective (Thorlabs) resulting in a lateral resolution of 8 μm. The specimen is then processed according to the standard procedure for a histological assessment by the pathology department. Hematoxylin-eosin (H&E) staining sections were prepared from the areas imaged with the OCT system. This allowed the OCT images to be compared and analyzed directly with the corresponding H&E sections regarding histopathological morphology.

Images of the histopathological findings were assigned to the OCT images in pseudonymized version. The OCT B-scans of the pancreatic lesions and tumors are then analyzed according to the findings of benign, premalignant (low risk, high risk) and malignant characteristic morphological features.

Given the exploratory nature of this study, no specific control measures or statistical analyses were performed due to the small sample size and pilot design. Instead, a qualitative assessment was conducted by comparing OCT images with corresponding histopathological findings.

## Results

The study cohort consisted of four patients, each contributing a distinct type of pancreatic tissue: white adipose tissue, intraductal papillary mucinous neoplasm (IPMN), pancreatic ductal adenocarcinoma (PDAC) based on IPMN, and neuroendocrine pancreatic carcinoma. The OCT images were taken back table in the operating room during surgery without the need to cut or process the tissue. By this way, the OCT images were available in the operating room within a few minutes.

**Fig. 1:**
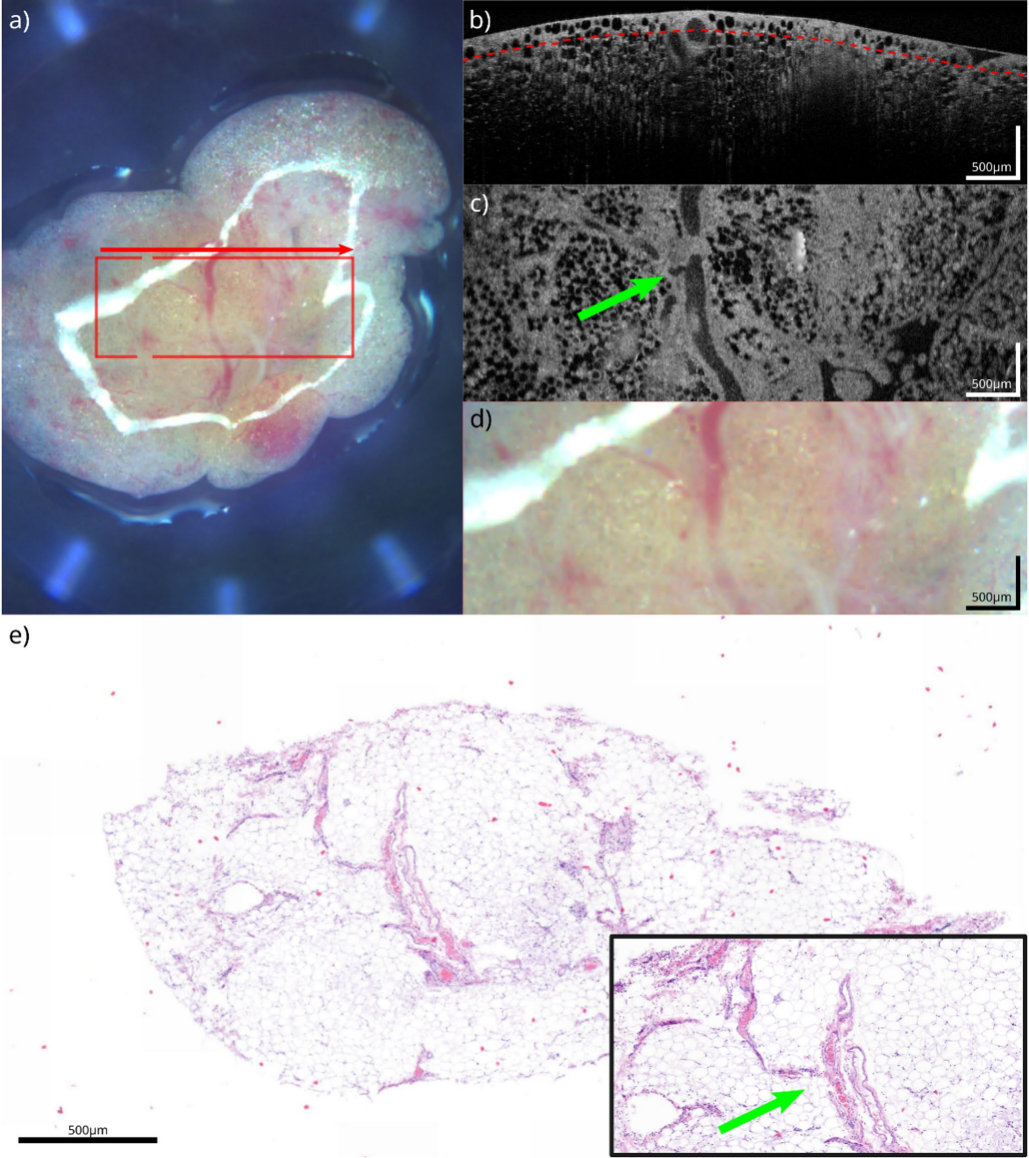
White adipose tissue: **a**) White light image of the specimen, showing the scanning area (red square) and scanning direction (red arrow). **b**) OCT B-scan from the acquired volume. **c**) Top-view slice extracted 120 µm below the tissue surface (red line in **b**). **d**) Detailed view of the scanning area, corresponding to (**c**). **e**) HE section of the specimen corresponding to (**c**) (detailed view bottom right). The green arrow highlights corresponding vessel structures between OCT and HE

**Fig. 2:**
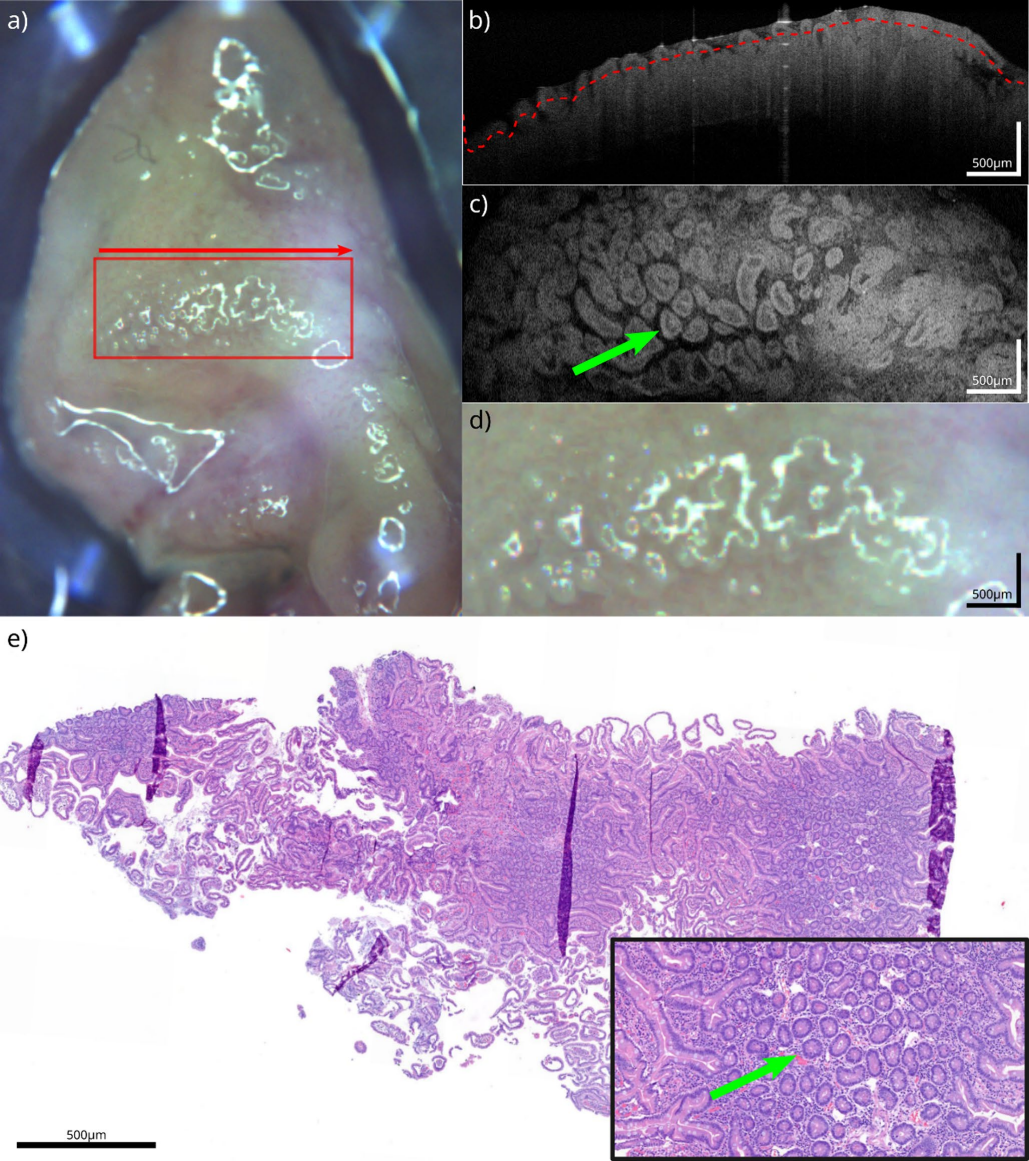
IPMN: **a**) White light image of the specimen, showing the scanning area (red square) and scanning direction (red arrow). **b**) OCT B-scan from the acquired volume. **c**) Top-view slice extracted 50 µm below the tissue surface (red line in **b**). **d**) Detailed view of the scanning area, corresponding to (**c**). **e**) HE section of the specimen corresponding to (**c**) (detailed view bottom right). The green arrow highlights groups of columnar mucin-producing cells visible in OCT and HE

**Fig. 3:**
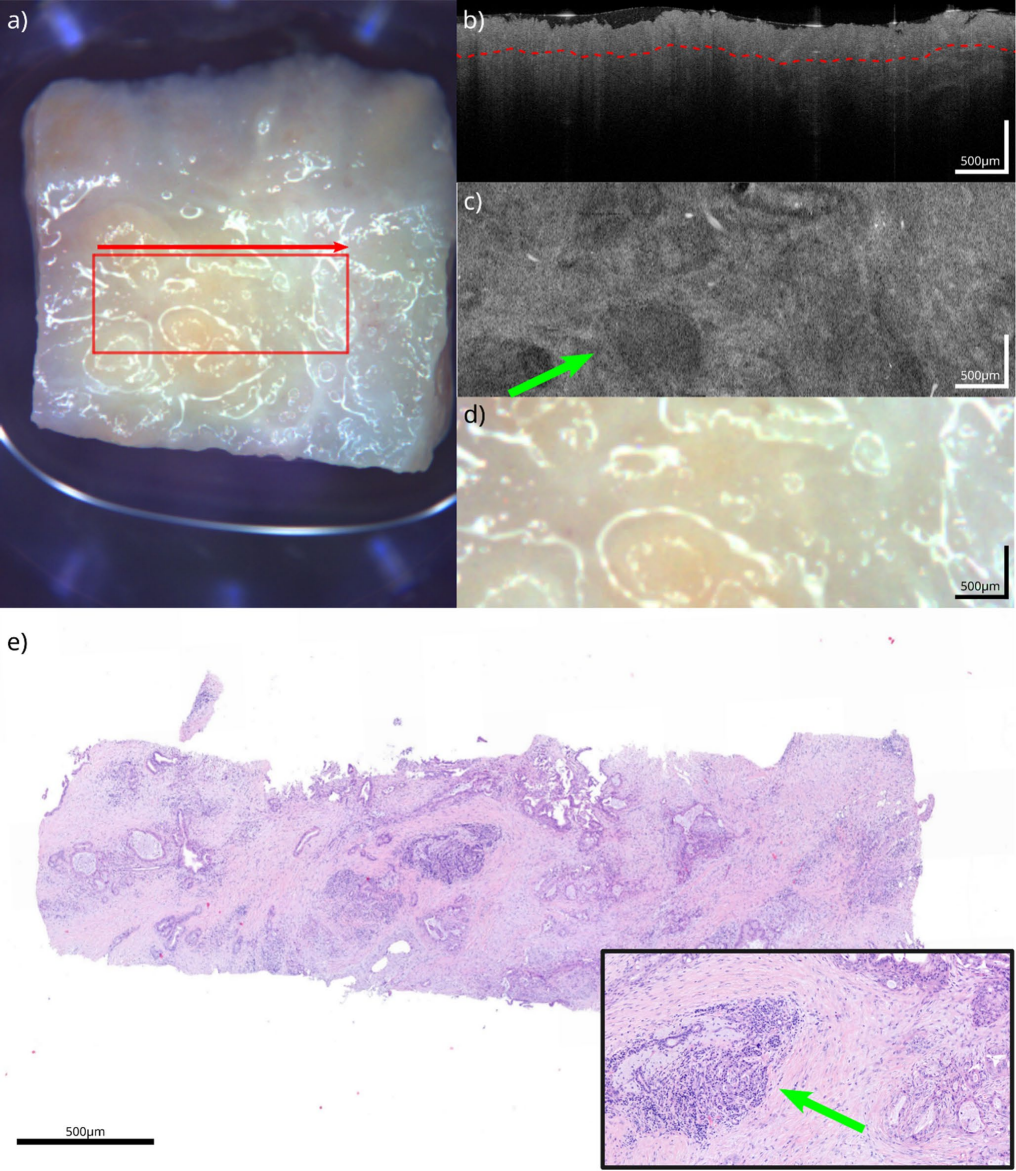
PDAC based on an IPMN: **a**) White light image of the specimen, showing the scanning area (red square) and scanning direction (red arrow). **b**) OCT B-scan from the acquired volume. **c**) Top-view slice extracted 240 µm below the tissue surface (red line in **b**). **d**) Detailed view of the scanning area, corresponding to (**c**). **e**) HE section of the specimen corresponding to (**b**) (detailed view bottom right). The green arrow highlights ductal adenocarcinoma with surrounding desmoplastic stromal response visible in OCT and HE

**Fig. 4:**
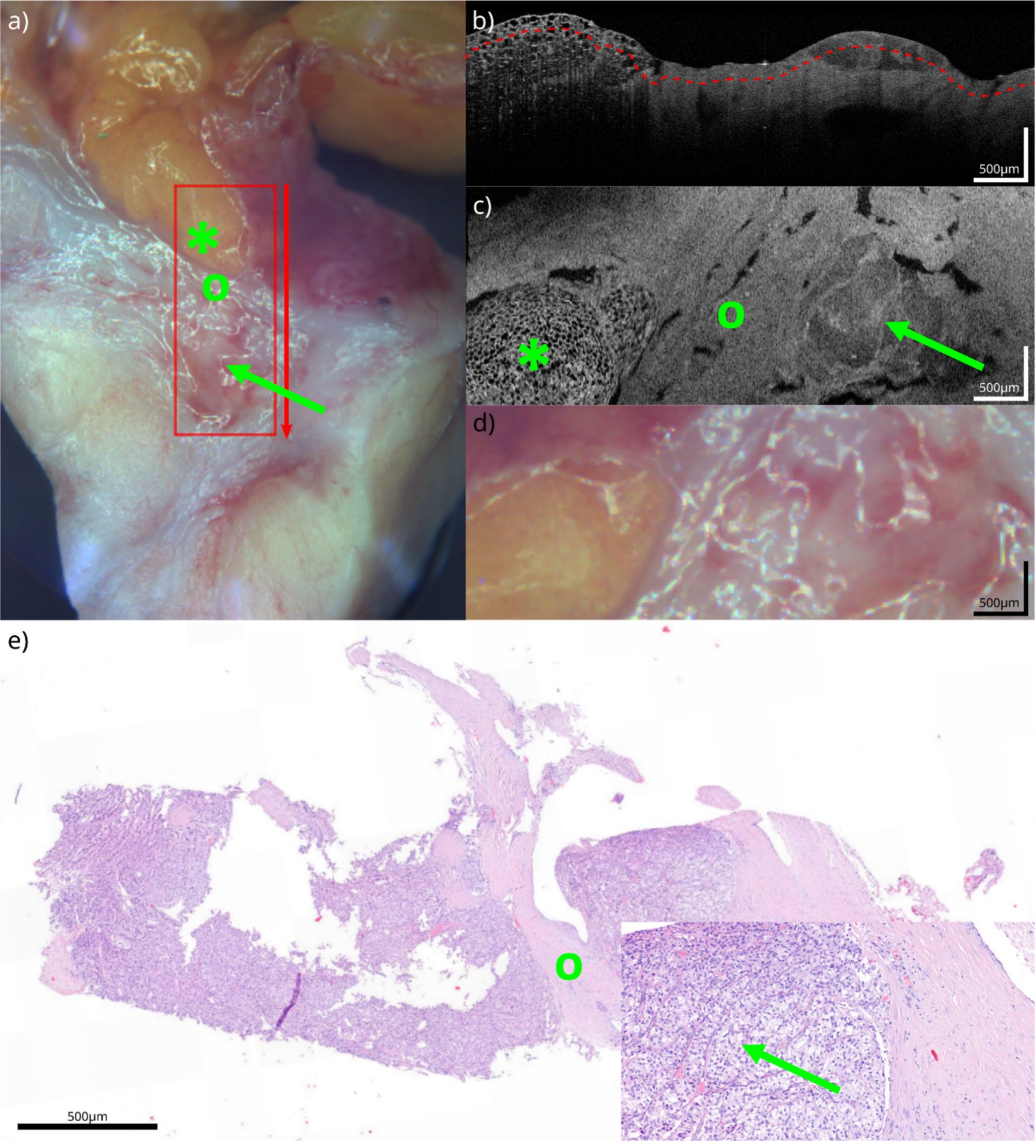
NET G2: **a**) White light image of the specimen, showing the scanning area (red square) and scanning direction (red arrow). **b**) OCT B-scan from the acquired volume. **c**) Top-view slice extracted 120 µm below the tissue surface (red line in **b**). **d**) Detailed view of the scanning area, corresponding to (**c**). **e**) HE section of the specimen (detailed view bottom right). The green arrow highlights NET with trabecular architecture visible in OCT and HE; *: fatty tissue; o: fibrotic tissue; arrow: NET

Macroscopic tissue samples with the microscopic findings and the corresponding OCT correlate are shown in Figs. 1, 2, 3 and 4. The samples presented are white fatty tissue (Fig. 1), an IPMN without worrisome features (Fig. 2), a moderately differentiated (G2) pancreatic ductal adenocarcinoma (PDAC) based on an IPMN (Fig. 3) and a neuroendocrine pancreatic carcinoma (Fig. 4). Both, OCT-images and histological samples are measured with 500 micrometer reference. The following examples demonstrate how OCT can detect key histological characteristics and tissue architecture.

Figure 1 shows conventional white adipose tissue in proximity to the pancreas. Macroscopically the sample impresses with a homogeneous, shiny, yellow surface with narrow fibrous septa in between (Fig. 1a/d). Large, homogenous spherical cells with a thin, crescent-shaped nucleus that is squeezed and pushed to the cell’s periphery by lipid droplets are the microscopic hallmarks of adipose tissue. A portion stained with standard HE shows distinct adipocytes. There are tiny membranes between cells (Fig. 1e). OCT reproduces the architecture of fat cells in detail. This clearly shows the homogeneous round structures with the narrow membranes between the fat cells (Fig. b/c) [14]. Additionally, OCT allows for visualization of the vascular system within the adipose tissue and even shows the content of bigger vessels.

Figure 2 represents an IPMN. Macroscopic impressions show an erased smooth surface, which is rough to touch (Fig. 2a/d). The intraductal proliferation of columnar mucin-producing cells, which can be of flat or papillary form. These range in size from microscopic folds to clearly apparent projections, what microscopically characterizes IPMNs. IPMNs are categorized as low-grade or high-grade depending on the epitheliums greatest level of cytoarchitectural atypia. Mild to moderate atypia is the hallmark of low-grade IPMNs, which may or may not exhibit papillary projections and mitoses (Fig. 1c/e). For comparison, severe atypia, papillae with irregular branching and budding, nuclear stratification with loss of polarity, pleomorphism, and conspicuous nucleoli are characteristics of high-grade IPMNs with many mitoses. Furthermore, OCT images replicate the columnar mucin-producing cells in their papillary projection (Fig. 2b/c) [14].

Figure 3 shows a moderately differentiated (G2) PDAC based on an IPMN. Macroscopic impressions are similar to the IPMN (Fig. 2a/d) with an erased smooth surface, which is rough to touch (Fig. 3a/d). However, microscopic architecture of the sample and the correlating OCT-image show similar characteristics. The majority of ductal adenocarcinomas are well to moderately differentiated, forming glandular structures resembling ducts that randomly invade the parenchyma of the pancreas and induce a potent desmoplastic stromal response (differentiation to IPMN, Fig. 2). The duct-like structures in well-differentiated carcinomas can be difficult to identify as neoplastic glands because they appear next to glands with characteristics more indicative of adenocarcinoma, such as angularly contoured ducts, branching, ruptured glands, and/or multilayered papillary epithelium with cribriform patterns. The most distinctive feature are burst glands that have cellular stroma partially lining them and mucus leaking out of them (Fig. 3c/e). OCT can visualize the densely elevated brighter desmoplastic stroma in particular. In addition, the darker islets can be distinguished from glandular structures that resemble ducts (Fig. 3b/c) [14]

An intermediately differentiated (G2) pancreatic neuroendocrine tumor (pNET) is illustrated in Fig. 4. Again, macroscopic impressions are similar to the IPMN and PDAC (Figs. 2 and 3 a/d) with an erased smooth surface, which is rough to touch (Fig. 4a/d). pNET (G2) has intermediate microscopic features, consisting of cells with mild atypia, organoid patterns, and no necrosis. Trabecular architecture, displaying an intermediate level of differentiation. There is also a proportion of fatty tissue in the sample that can be differentiated in both the H&E section and the OCT image. (Fig. 4c/e (*)). In the OCT images, the NET is visible due to its trabecular structure with an overall loosened architecture (Fig. 4c) [14].

## Discussion

In this exploratory study, we have shown that high-resolution OCT imaging can be used to differentiate between benign, premalignant, and malignant pancreatic pathologies in resected pancreatic specimens in the operating room. High-resolution OCT imaging thus has the potential to provide the surgeon with information on the dignity of pancreatic pathologies during surgery within a few minutes. There are several potential applications of this technology. First, this method of intraoperative OCT imaging could in perspective replace intraoperative frozen section examination of the dignity of pancreatic lesions and the resection margins during surgery. Second intraoperative application by OCT imaging via an endoscope [15], laparoscopic or robotic camera system might enable the surgeon to define the extent of resection (i.e. radical oncologic resection vs. local limited resection). Third, OCT technology provides digital data on the histological properties of the tissue. This data could be analyzed in perspective vie an artificial intelligence (AI) tool and provide the surgeon with real-time information about the pancreatic pathology during the operation.

Our findings align with previous studies that have demonstrated OCT’s ability to differentiate pancreatic pathologies. For instance, Iftimia et al. [1] showed that OCT could detect morphologic features of pancreatic cysts ex vivo, similar to our identification of papillary projections in IPMN and desmoplastic stroma in PDAC. Additionally, our results are consistent with studies on other tissues, such as Hsiung et al. [3], who used OCT to visualize tissue architecture in breast lesions, suggesting parallels in its applicability to pancreatic assessment. Prior work by Das et al. [9] and Testoni et al. [11] also supports OCT’s high concordance with histopathology in adenocarcinoma cases, further underscoring its diagnostic potential. Overall, there is currently scarce data in the international literature on the use of OCT methodology for the diagnostic differentiation of pancreatic lesions [1, 7–9, 15, 16]. However, the limited data available underline our findings that OCT can improve the diagnosis of pathological findings of the pancreato-biliary system [9, 10]. In the presence of adenocarcinoma, initial studies have shown that OCT images are 100% concordant with final histopathologic findings in sections with adenocarcinoma [11]. Further data suggest that OCT methodology has the potential to reveal specific morphologic features of pancreatic cysts. The sensitivity and specificity for the differentiation of cystic pancreatic space-occupying lesions was 95% in the initial studies [1, 12].

The interpretation of OCT images requires a high level of experience and training, similar to the conventional histopathologic examination of pancreatic specimens, AI supported imaging requires large amounts of data for AI learning purposes.

Our study is unique in its exploration of a range of pancreatic pathologies, including both cystic and solid lesions, within a surgical setting. This adds to the growing body of evidence that OCT could enhance intraoperative decision-making by providing real-time, high-resolution imaging. The clinical implications are significant: OCT could potentially replace frozen section analysis or guide the extent of resection, thereby improving surgical precision and patient outcomes. Considering intraoperative resection margin assessment, there are no limitations of re-evaluation if there may be uncertainty of the R0 siuation.

As a limitation, it must be emphasized that in this current study, we only explored the possibility of assessing pancreatic morphology using OCT imaging. The small study population limits the generalizability of these findings, and future studies with larger cohorts are necessary to validate the clinical utility of OCT in this context. However, exploratory studies are needed to investigate the potential of OCT imaging for specific clinical issues such as the assessment of the dignity of pancreatic cystic neoplasms or the resection margin. OCT imaging has a certain limitation in the resolution of pathologies located in the depth of the pancreas, as the penetration depth is limited to up to 7–8 mm. A solution could be a combination of endoscopic OCT assessment via the pancreatic duct in combination with laparoscopy. As previously mentioned in a next step, a special in vivo OCT laparoscope with real-time volumetric data acquisition capabilities [14] could be used to assess the pancreatic tissue in vivo prior to resection as part of a surgical exploration of the pancreas. Such in vivo OCT imaging could potentially allow a more precise intraoperative assessment between benign, premalignant or malignant pancreatic pathologies prior to resection. This approach therefore has the potential to increase precision in pancreatic surgery or to prevent unnecessary extended oncological resection in benign or premalignant pancreatic pathologies.

## Conclusion

In conclusion, OCT imaging has the potential to morphologically differentiate between benign, pre-malignant and malignant pancreatic pathologies. The application of OCT imaging enables morphologic examination of pancreatic lesions at a histopathologic level within a few minutes during surgery, in this case back table in the operating room, and could thus enable time-saving and appropriate treatment decisions in the operating room. As this study was purely exploratory, further studies are needed to validate the accuracy of the technique in differentiating pancreatic lesions, particularly with regard to intraoperative clinical decision making.

## Data Availability

No datasets were generated or analysed during the current study.

## References

[1] Iftimia N, Cizginer S, Deshpande V, Pitman M, Tatli S, Iftimia NA et al (2011) Differentiation of pancreatic cysts with optical coherence tomography (OCT) imaging: an ex vivo pilot study. Biomed Opt Express 2(8):2372 21833374 10.1364/BOE.2.002372 PMC3149535

[2] Sahani DV, Miller JC, Fernàndez del Castillo C, Brugge WR, Thrall JH, Lee SI (2009) Cystic pancreatic lesions: classification and management. J Am Coll Radiol 6(5):376–380 19394581 10.1016/j.jacr.2008.10.004

[3] Hsiung PL, Phatak DR, Chen Y, Aguirre AD, Fujimoto JG, Connolly JL (2007) Benign and malignant lesions in the human breast depicted with ultrahigh resolution and Three-dimensional optical coherence tomography. Radiology 244(3):865–874 17630358 10.1148/radiol.2443061536

[4] Ellebrecht DB, Latus S, Schlaefer A, Keck T, Gessert N (2020) Towards an optical biopsy during visceral surgical interventions. Visc Med 36(2):70–79 32355663 10.1159/000505938 PMC7184834

[5] Federle MP, McGrath KM (2007) Cystic neoplasms of the pancreas. Gastroenterol Clin N Am 36(2):365–376 10.1016/j.gtc.2007.03.014 17533084

[6] Wargo JA, Fernandez-del-Castillo C, Warshaw AL (2009) Management of pancreatic serous cystadenomas. Adv Surg 43(1):23–34 19845167 10.1016/j.yasu.2009.03.001

[7] Abraham AS, Simon B, Eapen A, Sathyakumar K, Chandramohan A, Raju RS et al (2020) Role of Cross-sectional imaging (CT/MRI) in characterization and distinguishing benign from malignant/potentially malignant cystic lesions of pancreas. JCIS 10:28 32494507 10.25259/JCIS_15_2020 PMC7265468

[8] Mahmud MS (2013) Imaging pancreatobiliary ductal system with optical coherence tomography: A review. WJGE 5(11):540 24255746 10.4253/wjge.v5.i11.540 PMC3831196

[9] Das A, Sivak MV, Chak A, Wong RC, Westphal V, Rollins AM et al (2001) High-resolution endoscopic imaging of the GI tract: a comparative study of optical coherence tomography versus high-frequency catheter probe EUS. Gastrointest Endosc 54(2):219–224 11474394 10.1067/mge.2001.116109

[10] Arvanitakis M, Hookey L, Tessier G, Demetter P, Nagy N, Stellke A et al (2009) Intraductal optical coherence tomography during endoscopic retrograde cholangiopancreatography for investigation of biliary strictures. Endoscopy 41(8):696–701 19618343 10.1055/s-0029-1214950

[11] Testoni PA, Mariani A, Mangiavillano B, Arcidiacono PG, Di Pietro S, Masci E (2007) Intraductal optical coherence tomography for investigating main pancreatic duct strictures. Am J Gastroenterol 102(2):269–274 17100970 10.1111/j.1572-0241.2006.00940.x

[12] Cizginer S, Deshpande V, Iftimia N, Karaca C, Brugge WR (2009) 233 optical coherence tomography (OCT) imaging can detect fine morphologic features of pancreatic cystic neoplasms and differentiate between mucinous and Non-Mucinous cysts. Gastroenterology 136(5):A–45

[13] Huang D, Swanson EA, Lin CP, Schuman JS, Stinson WG, Chang W et al (1991) Opt Coherence Tomography Sci 254(5035):1178–1181 10.1126/science.1957169 PMC4638169 1957169

[14] Nagtegaal ID, Odze RD, Klimstra D, Paradis V, Rugge M, Schirmacher P et al (2020) The 2019 WHO classification of tumours of the digestive system. Histopathology 76(2):182–188 31433515 10.1111/his.13975 PMC7003895

[15] Lu L, Hu Z, Frankel W, Shen R, Chen W, Pan X et al (2021) Using endoscopic optical coherence tomography to detect and treat Early-Stage pancreatic cancers. Front Oncol 11:591484 33791200 10.3389/fonc.2021.591484 PMC8005647

[16] Ahmad NA, Kochman ML, Brensinger C, Brugge WR, Faigel DO, Gress FG et al (2003) Interobserver agreement among endosonographers for the diagnosis of neoplastic versus non-neoplastic pancreatic cystic lesions. Gastrointest Endosc 58(1):59–64 12838222 10.1067/mge.2003.298

